# Materia Medica and Therapeutics

**Published:** 1857-11

**Authors:** 


ART. XIV.—*Materia Medica and Therapeutics, with ample Illustrations of Practice in all the Departments of Medical Science, and very copious Notes of Toxicology, suited to the wants of Medical Students, Practitioners, and Teachers.* A new Edition, revised and enlarged. By THOMAS D. MITCHELL, A. M., M. D., Professor of Materia Medica and General Therapeutics, in Jefferson Medical College, and formerly Professor of the same in Transylvania University. Philadelphia: J. B. Lippincott & Co.; pp. 820, octavo. 1857.

A QUOTATION from the preface to this edition is all that need be given in this place, at this time. "It is written to say, that the work has been carefully revised and brought down to the present time. As a consequence, the bulk of the volume is augmented by eighty-two pages, a circumstance altogether unavoidable. The author has added his own experience on various points, and given the best testimony of the great world of physic, with the design of putting a book of real value into the hands of students and practitioners. It is believed that every valuable new application of an old remedy, as well as the desirable uses of agents claimed to be new, down to the date of this prefatory note, are here presented so as to set forth their real or apparent worth." A more extended notice of Professor Mitchell's work will appear hereafter. Meanwhile, we bespeak for it the favorable notice of the profession.

